# A prospective randomized study of conservative versus surgical treatment of unstable palmar plate disruption in the proximal interphalangeal finger joint

**DOI:** 10.1007/s11751-013-0154-y

**Published:** 2013-02-23

**Authors:** Jens Christian Werlinrud, Kirstin Petersen, Jens Lauritsen, Søren Larsen, Henrik Schrøder

**Affiliations:** Department Of Orthopedics, Odense University Hospital, Kristiansdals Allé 162, DK-5250 Odense SV, Region of Southern Denmark

**Keywords:** Conservative versus surgical intervention, Hyperextension injury, Palmar ligament, PIP, Prospective randomized study, Proximal interphalangeal joint, Palmar fibrocartilage, Palmar plate

## Abstract

The aim of this study was to assess the effect of conservative versus operative treatment for unstable palmar plate disruption in the proximal interphalangeal (PIP) joint of the fingers with respect to preservation of joint stability, mobility, and pain. The study was conducted as a prospective study in which 83 patients were randomly assigned into 2 groups: (1) conservative treatment with a rigid splint for 2 weeks, (2) surgical reattachment of the palmar plate in local anesthesia followed by 2 weeks of immobilization in a plaster cast. Both groups were thereafter treated by taping to the neighboring finger for 3 weeks. With regard to hyperextension instability, stiffness, and pain, there is no significant difference in outcome between patients with traumatic palmar plate lesions and hyperextension instability treated with surgical repair and patients treated conservatively with a splint. We do not recommend primary surgical repair of unstable isolated palmar plate lesions in the proximal interphalangeal joints of the 4 ulnar fingers.

*Type of study*/*level of evidence* Therapeutic, Level II.

## Introduction

Acute hyperextension injuries of the fingers are common. The joint most often affected is the proximal interphalangeal (PIP) joint, and the most common causes of injury are ball sport [1, 2] and fall accidents [3, 4]. Most often we find the joint to be stable, and the injury is treated as a sprain. A small palmar lip avulsion is commonly seen as part of a total or partial palmar plate rupture. Various complications to hyperextension injuries to the PIP joint have been described and may include pain, reduced range of motion, and hyperextension laxity.

When reviewing the literature, we found no study investigating the evidence for surgical versus conservative treatment of unstable palmar plate disruptions without fracture or dorsal subluxation. We also found no study advocating surgical repair of palmar plate ruptures in the four ulnar fingers. Quite a lot of studies advocate conservative treatment of palmar plate injuries and a few of these are cited in this article. Our study assesses patients with isolated hyperextension laxity following an acute injury to one of the four ulnar PIP joints of the fingers. Patients with isolated hyperextension laxity and a small distal osseous avulsion, as part of the isolated palmar plate rupture, were also included. By performing a randomized controlled study, we compare surgical versus conservative treatment of this injury by 3 outcome results: stability, range of motion, and pain. The study compares the effect of surgical repair of the palmar plate compared to conservative treatment only.

## Materials and methods

Patients were included in the time from April 01, 2000, to September 12, 2006, and the follow-up period spanned from July 01, 2000, to September 12, 2007.

The study was conducted in accordance with the Helsinki II declaration and was approved by the Scientific Ethics Committee in Fyn and Vejle Counties.

Patients included were residents of Odense city and surrounding areas on the island of Fyn. The population in this area is 484.000, and the annual number of patients seen in the emergency room is approximately 33.000 [5]. Of all patients with distorted fingers caused by sports 45 % were caused by playing handball and 24 % by playing soccer [5].

Patients seen in the emergency room at Odense University Hospital with a suspected palmar plate disruption were reexamined the following day by a hand surgeon. Patients were included and randomized if the symptoms and objective findings were found to match the criteria’s for inclusion. No primary visitation was done before arrival in the emergency room.

Randomization was done by randomly selecting from a mixed box of closed envelopes. Conservative treatment was initiated at this point. Patients randomized for surgical intervention were booked for surgery as soon as possible by the surgeon on duty.

By a power calculation, we concluded that a total number of 80 (40 + 40) patients for inclusion would be sufficient.

The sample size was based on an expected difference in treatment outcome of 25 %, a significance level of 5 %, and a 10 % risk of type 2 errors. No interim analysis or stopping rules were applied.

Group 1 was treated conservatively with a rigid splint for 2 weeks followed by taping to the neighboring finger for 3 weeks.

Group 2 was treated with surgical reattachment. Surgical repair of the palmar plate was done as a bloodless procedure in local anesthesia. A standard palmar zigzag incision in the skin was used, and the palmar plates sutured in both sides with a Ticron 4–0 suture. Small osseous avulsions at the base of the middle phalanx were reduced and fixated with Ticron 4–0 suture after drilling a small hole in the bone with a Kirschner-wire or hypodermic needle. The skin was closed with Ethilon 5–0 sutures. Sutures were removed along with the plaster cast 2 weeks postoperatively, and buddy taping was applied for the following 3 weeks. Rehabilitating exercises were initiated after cast removal.

### Inclusion criteria

Age >18, injury less than 4 days old, tenderness by clinical examination on the palmar aspect of the PIP joint and hyperextension instability. Hyperextension instability was defined as hyperextension laxity compared with the same finger on the opposite hand.

### Exclusion criteria’s

Former injury to the joint, inflammatory joint disease involving the injured joint, fracture in close proximity to the joint (except for avulsions involving less than 25 % of the joint surface), more than one finger injured, non-reducible dislocation of the joint, suspected interposition of tissue after reposition of a dislocated joint, side instability.

#### Post-treatment protocol

Patients in the study group were seen in our out-patient clinic at follow-up 3 and 12 months after time of injury. Measured outcome results were as follows: Range of motion (ROM) was measured with a ruler as fingertip-palmar distance at the distal palmar crease. Hyperextension stability—when compared to the opposite finger—was measured with a small protractor. Pain was evaluated using a visual analog scale (VAS). The assessment was done by consultant hand surgeons and resident trainees in orthopedic surgery. Surgeons evaluating the outcome results were instructed on a single meeting.

The data were evaluated statistically with an unpaired *t* test.

## Results

Eighty-eight patients were assigned to randomization into the 2 groups. Patients were between 18 and 79 years old with a mean age of 39 years. Sixty-five percent of the injuries were caused by sports and traffic-related injuries accounted for 11 %.

Five of 88 patients were excluded (see Table 1): 1 patient had no clinical signs of hyperextension instability on a secondary examination and 4 patients failed to appear.

**Table 1:** Site of injury

	Surgical repair	Conservative treatment	Total
Side			
Right hand	18 (53.3)	14 (43.8)	32 (100 %)
Left hand	23 (45.1)	28 (54.9)	51 (100 %)
	41 (49.4)	42 (50.6)	83 (100 %)
Injured finger			
2nd	4 (44.4 %)	5 (55.6 %)	9 (100 %)
3rd	11 (52.4 %)	10 (47.6 %)	21 (100 %)
4th	12 (52.2 %)	11 (47.8 %)	23 (100 %)
5th	14 (46.7 %)	16 (53.3 %)	30 (100 %)
Total	41 (49.4 %)	42 (50.6 %)	83

Forty-one patients were randomized to surgery (25 men, 16 women) and 42 to conservative treatment (20 men, 22 women). All patients were seen primarily at the emergency room and at follow-up as outpatients.

With regard to our three primary endpoints, pain, stability, and range of motion, we found no significant difference between the 2 groups on evaluation 12 months after time of injury, see Table 2. At 12-month follow-up, patients were divided into groups of more than 5, 15, and 25 degrees of hyperextension instability. There was no significant difference in the number of patients treated surgically or conservatively in these three groups. Almost twice the numbers of patients in the group treated conservatively were found to have a hyperextension laxity of up to 25 degrees, but the numbers are not statistically significant.

**Table 2:** Primary endpoints

Pain on a VAS from 1–10	N	Mean VAS-score	95 % CI
Surgical group	41	3.50	1.31–5.68
Conservative group	42	3.04	0.39–5.69

Instability	Total	Unstable on extension	%	95 % CI
5° instability on extension				
Total	83	21	25.3	17.2–35.6
Surgery	41	7	17.1	8.5–31.3
Conserv	42	14	33.3	21.0–48.4
15° instability on extension				
Total	83	13	15.7	9.4–25.0
Surgery	41	5	12.2	5.3–25.5
Conserv	42	8	19.0	10.0–33.3
25° instability on extension				
Total	83	2	2.4	0.7–8.4
Surgery	41	1	2.4	0.4–12.6
Conserv	42	1	2.4	0.4–12.3

Pulp-to-palm distance		Mean distance (mm)	95 % Confidence interval
Surgical group	41	0.171	0.3327–0.0145
Conservative group	42	0.0952	0.188–0.0027

After 12 months, we found that 15/41 (37 %) in the group of surgically treated patients experienced complications, see Table 3. Four of these were cosmetic and had no bearing on the final result. Nine patients experienced cold intolerance and 8 experienced dysesthesia after 12 months. It is not reported whether there was an improvement in these complications after the 12 months of follow-up.

**Table 3:** Complications

Complications	Surgical group	Conservative treatment
Yes	15 (36.6 %)	(0)
No	26 (63.4 %)	(0)
Total	41 (100 %)	(0)

Complaints	Surgery	Conservative
Cold intolerance	9	0
Dysesthesia	8	0
Cosmetic	4	0

## Discussion

A substantial proportion of patients, who were treated by surgical reattachment, experienced complications, whereas there were no complications in the group treated conservatively. Arguably, our result may have differed if other regimens of treatment had been applied. For example, in the conservative group, we chose a rigid splint instead of a dynamic splint. With regard to the surgically treated group, the palmar plate reattachment was done by the surgeon on duty—not necessarily a hand surgeon. Had it been an experienced hand surgeon performing the repair, results may have differed, and our study might also have been more reproducible if all patients were treated by the same surgeon. Alas, such settings are not representative of everyday procedures in a Danish orthopedic department. We have not reported the incidence of flexion contracture, a complication more common than hyperextension laxity [6].

Our setting represents standard procedures in most countries with regard to both selection of patient material and treatment method.

Many aspects on palmar plate injuries have been discussed. As described by Bowers [7], the anatomical site of rupture has been the cause of much debate, but it is now generally accepted that the site of injury is virtually always at the distal palmar plate—bone junction. The mechanism itself is elegantly illustrated under experimental conditions by Hintringer [8].

In a clinical and radiological follow-up study of 155 patients with injuries to the PIP joint in the middle finger, Höcker and Menschik [1] found that 82 % of the patients had a small avulsion fracture on the palmar lip of the middle phalanx −97 % with no significant dislocation. Ninety-nine percent of the patients were treated conservatively with splinting, most often 3 weeks. With regard to extension deficits, there was no significant difference between splinting for 2 and 3 weeks. Ninety-five percent had good or excellent results. On follow-up, 9 % had hyperextension laxity, but this was reported to be without clinical significance for the patients.

Leibovis and Bowers [9] do not recommend splinting in extension because of the risk of hyperextension laxity. Bowers argues that immobilization should be in a semi-flexed position in order to avoid hyperextension deformity [10], but Incavo et al. [6] did a retrospective review on 22 patients with stable hyperextension injuries to the PIP joint with small avulsion fractures from the base of the proximal phalanx. They concluded that 7–10 days of immobilization in full extension followed by buddy taping for 3 weeks and active range of motion was recommendable since none of the patients developed hyperextension laxity. Thomsen et al. [11] compared aluminum splinting versus elastic double-finger bandage in patients with isolated hyperextension injuries and found no difference in outcome and all patients had satisfying results (82 % had excellent results).

Reviewing the literature we found various recommendations for conservative treatment of palmar plate lesions. We found no papers recommending surgical repair of unstable isolated palmar plate lesions without fracture. Our results support the current literature recommending conservative treatment of unstable isolated palmar plate lesions.
